# Supplementary material for: Congenital transmission of Chagas disease: The role of newborn therapy on the disease’s dynamics
Source: PLoS One. 2024 Sep 19;19(9):e0308136. doi: 10.1371/journal.pone.0308136 (PMC11412536; doi:10.1371/journal.pone.0308136)
Supplement: Attachment — Submitted filename: Response to Reviewers.docx [file pone.0308136.s001.docx]

Dear Editor:

The authors would like to thank you for the opportunity to revise our manuscript. We also want to thank both reviewers and the Handling Editor for thoughtful comments and suggestions on the previous version of this manuscript. We have thoroughly revised it and addressed their comments. Below (in blue), please find our response to the reviewers' comments.

**REVIEWER 1:**

We appreciate the reviewer's feedback and do address the concerns below.

 This is a relatively standard ODE model of a disease. The model is appropriate for Chagas disease and the analysis is sound.

The biggest issue with this paper lies in the result section. The results and the figures are presented in a cumbersome manner. Results on the effect of alpha should be presented in a table and also a figure (with change of alpha on the x axis and the effect on the y axis)
We thank the reviewer for this comment. We have summarized the impact of the alpha on the infected individual's compartment of the model in Table 3. Furthermore, we have added two new plots: Figure 2(a) (contour plot), and Figure 2(b) (I^*-vs alpha) to further demonstrate the effect of alpha on the model dynamics.

An even more important issue is that the authors consider only DFE and not endemic equilibrium. Their proofs for stability of DFE are standard and can perhaps be streamlined. The formulas for EE are missing completely and should be added.

We thank the reviewer for this comment. We have streamlined the local stability proof of DFE.

The validation of the model is missing, i.e., there is no clear reason to believe that the model fits reality. In fact, the model most likely does not fit the reality as it predicts an exponential growth. I would be inclined to believe that in most regions with Chagas, the disease is already endemic and more or less on a stable levels (or fluctuating due to environmental and other changes) but not exponentially growing.

Due to the above issue, the EE should be evaluated, the model validated, perhaps compared to EE and then the sensitivity to alpha reconsidered (i.e. describe how alpha influences the EE, not the growth)

We thank the reviewer for this comment. This is an ODE model that grows, decays, and reaches an equilibrium under certain conditions. We have evaluated and derived conditions for which the EE exists. We have also analyzed the impact of the alpha on the EE both numerically and theoretically. Figure 5(b) (I^*-vs alpha) demonstrates the effect of alpha on the EE and these results collaborate.

**REVIEWER 2:**

We thank the reviewer for carefully reading the manuscript and for valuable suggestions. We have incorporated the suggestions in the revised version.

Dear authors, this is a study highlighting the importance of adressing the importance of newborn therapy for Chagas Disease control. The methods are sound and the results interesting. There are but a few comments I would like to do.
1. I would recommend abbreviating the introduction.

We thank the reviewer for this comment. We agree with the reviewer that the introduction was long and have reduced it by one paragraph.

2. I would add abbreviation definitions to the footnote of figure 1.

We thank the reviewer for this comment. However, the comment is not clear to us. Figure I is the schematic diagram of the model. The model parameters are defined in Table 1 and the variables are defined in the first paragraph of the materials and methods section.

3. I would end the introduction section stating the study question.

We thank the reviewer for this comment. We have rephrased the last paragraph of the introduction and added the research question.

4. My biggest concern, however, is the fact that treatment of infected humans with Chronic Chagas Disease has not been included in the model. I would think this is very likely to have an important impact on the congenital transmission for two reasons, more infected mothers will be treated and thus would not transmit the disease and because as less infected humans are present less infections of the bugs would occur. I believe this is an important caveat and should be addressed in the discussion.

We thank the reviewer for this comment. Several studies have confirmed the effectiveness of acutely infected individuals and are less effective for chronic Chagas disease patients. Nevertheless, we have addressed this comment as a limitation.
5. I also miss a section evaluating the limitations and strengths of the conclusions reached

We thank the reviewer for this comment. We have added a paragraph addressing limitations and strengths.
