# Supplementary material for: Congenital transmission of Chagas disease: The role of newborn therapy on the disease’s dynamics
Source: PLoS One. 2024 Sep 19;19(9):e0308136. doi: 10.1371/journal.pone.0308136 (PMC11412536; doi:10.1371/journal.pone.0308136)
Supplement: Attachment — Submitted filename: Response to Reviewers_2.docx [file pone.0308136.s002.docx]

Dear Editor:

The authors would like to thank you again for the opportunity to revise our manuscript. We also want to thank the reviewer for thoughtful comments and suggestions on the previous version of this manuscript. We have thoroughly revised it and addressed their comments. Below (in blue), please find our response to the reviewers' comments.

**REVIEWER 1:**

Reviewer #1: Thank you for revising the manuscript. There are still several issues that should be addressed

1) The authors provide formulas for the endemic equilibrium but not the derivation. Some calculations should be provided that would indicate that the formulas are correct. It is common that the formulas for EE contains basic reproduction number and that one needs the reproduction number to be greater than 1 for the formulas to be biologically reasonable. The provided formulas are sort of along the lines, but it is really not clear that they are correct. So, my suggestion is to a) include the calculations and b) look for the reproduction number in the formulas.

We thank the reviewer for this comment and suggestion. We have added some details and streamlined the derivation of the EE. We also agree with the reviewer that the EE of some models can be expressed in terms of the reproduction number; unfortunately, our EE cannot be written in terms of our R_c/R_L.

2) The figures still show exponential growth (i.e. what happens when one varies alpha in the DFE). As mentioned earlier, the diseases is endemic. So it would make more sense to investigate what happens when one varies alpha in EE.

We thank the reviewer for the comment. We have provided simulations showing the effects of alpha on the system at EE (Figure 4) and DFE (Figure 5).

in light of the point 3 below, if the authors indeed change the model, this point be addressed by varying r rather than alpha (assuming omega is more or less given by the nature of the treatment).


3) upon closer reading of the model description, there appears to be a very important and significant inconsistency in how authors treat alpha and p and r. They introduce alpha as omega times r where omega is treatment efficacy and r is a treatment rate. That means that alpha is a rate. The term alpha times p times M for the transmission from M to Sh does not make much sense (one cannot multiply rate with a rate to get a rate). Similarly, the term (1-alpha) in the flow from M to Ia does not make much sense either (on its own and definitely not when multiplied again by a rate p). It seems that much more natural rates from M to Sh would be omega times r and from M to Ia to be (1-omega) times r. There is also probably another process (such as aging) during which the untreated newborns from M become Ia. This is likely why authors used p in the model (and the overall rate from M to Ia would then be p+(1-omega)r).
So, my suggestions is for the authors to revise the model appropriately (or if they do not agree with the above interpretation, explain the model properly)

We thank the reviewer for this comment. This is important feedback, and we agree that the parameter alpha was not well-defined. We have redefined and explained alpha. We also thank the reviewer for the suggested model. It is a great idea, and we thoroughly explored it. However, we observed some concerns and caveats in this version of the model. For example, while I_a decreases with respect to omega (efficacy), it increases with respect to the treatment rate, r, with a fixed omega.
